# Evaluation of RT-LAMP for SARS-CoV-2 Detection in Animal Feces

**DOI:** 10.3390/v17060783

**Published:** 2025-05-29

**Authors:** Aimee Pepper, Sandipty Kayastha, Megan Miller, Jake Guag, Andriy Tkachenko, Matthew Allender, Karen Terio, Leyi Wang

**Affiliations:** 1 Veterinary Diagnostic Laboratory, Department of Veterinary Clinical Medicine, University of Illinois College of Veterinary Medicine, Urbana, IL 61802, USA; apepper2@illinois.edu (A.P.);; 2 Center for Veterinary Medicine, U.S. Food and Drug Administration, Laurel, MD 20708, USA; 3 Zoological Pathology Program, University of Illinois College of Veterinary Medicine, Brookfield, IL 60513, USA

**Keywords:** SARS-CoV-2, RT-LAMP, rRT-PCR, feces, animals

## Abstract

The wide host range, potential lethality, and zoonotic potential of SARS-CoV-2 infection in animals highlights the need for additional surveillance strategies. We validated a commercial, pH-based, colorimetric RT-LAMP assay for the detection of SARS-CoV-2 RNA in animal feces. The comparator assay was rRT-PCR. The limit of detection of the RT-LAMP assay was 72 genome copies per reaction. RT-LAMP was highly specific for SARS-CoV-2 and did not detect other human or animal coronaviruses. RT-LAMP was robust, with valid results generated for incubation lengths of 30 to 45 min, incubation temperatures of 60 to 70 °C, and reaction volumes of 10 to 25 µL. The diagnostic sensitivity was 100% for clinical fecal samples with high viral loads (Ct ≤ 25), 97.4% for samples with moderate to high viral loads (Ct ≤ 33), and 62% overall (Ct ≤ 40). The diagnostic specificity was 97.9%. Blinded method testing organized by an independent laboratory confirmed the satisfactory reproducibility of the assay. To our knowledge, this study represents the first validation of RT-LAMP for SARS-CoV-2 detection in animals. RT-LAMP testing could detect SARS-CoV-2 infection more rapidly and at the point of care in animals with moderate to high viral loads, allowing for earlier implementation of control measures.

## 1. Introduction

Severe acute respiratory syndrome coronavirus 2 (SARS-CoV-2), the causative agent of the coronavirus disease 19 (COVID-19) pandemic, is an enveloped, positive-sense, single-stranded RNA virus belonging to the order *Nidovirales*, family *Coronaviridae*, subfamily *Orthocoronaviridae*, genus *Betacoronavirus*, and subgenus *Sarbecovirus* [1]. SARS-CoV-2, initially termed 2019-nCoV, was first discovered in December 2019 in association with a cluster of pneumonia cases in human patients that were epidemiologically linked to the Huanan seafood and live animal wholesale market in Wuhan, Hubei Province, China [2]. Since then, numerous SARS-CoV-2 genetic variants have emerged, including variants of concern Alpha, Beta, Gamma, Delta, and Omicron, with Omicron sublineages still in circulation [3]. At the time of this study, 777 million cases of COVID-19 have been reported worldwide, with 7 million total human deaths [4].

To date, natural infection with SARS-CoV-2 has been reported in at least 40 different mammalian species, including carnivores of the families Felidae, Mustelidae, Canidae, Procyonidae, Hyaenidae, and Viverridae; artiodactyls of the families Cervidae, Bovidae, and Hippopotamidae; primates of the families Atelidae, Hominidae, Callitrichidae, Cebidae, and Cercopithecidae; rodents of the families Cricetidae, Castoridae, Muridae, and Sciuridae; bats of the family Vespertilionidae; armadillos of the family Chlamyphoridae; lagomorphs of the family Leporidae; marsupials of the family Didelphidae; perissodactyls of the family Equidae; anteaters of the family Myrmecophagidae; and sirenians of the family Trichechidae [5]. Animals infected with SARS-CoV-2 commonly present with mild respiratory and/or gastrointestinal signs, though subclinical infection or even fulminant disease leading to death can occur [5,6]. SARS-CoV-2 infection is particularly devastating in farmed mink (*Neovison vison*), which succumb to interstitial pneumonia in the form of diffuse alveolar damage (the histologic hallmark of acute respiratory distress syndrome), like humans with severe disease [7,8]. Snow leopards (*Panthera uncia*) also appear particularly susceptible to SARS-CoV-2 infection, particularly with the Delta variant, which can lead to a more prolonged disease course within the respiratory tract, with secondary bacterial and fungal infections leading to death [9]. The lethality of SARS-CoV-2 infection in certain animal species, such as mink and snow leopards, highlights the need for earlier detection of the virus such that control measures can be implemented to limit viral spread and thus animal mortality.

Reverse zoonotic (anthropozoonotic) spread of SARS-CoV-2 from humans to animals appears to be the main route of transmission for animal infection [6]. However, subsequent zoonotic transmission of SARS-CoV-2 from animals back to humans has previously been demonstrated in farmed mink (*Neovison vison*) [10,11], golden hamsters (*Mesocricetus auratus*) [12], one domestic cat (*Felis catus*) [13], one African lion (*Panthera leo*) [14], and white-tailed deer (*Odocoileus virginianus*) [15,16]. Of concern, in free-ranging white-tailed deer, multiple genetic lineages of SARS-CoV-2 have been shown to co-circulate, including variants of concern no longer circulating within human populations, implicating white-tailed deer as a potential animal reservoir of SARS-CoV-2 [16]. Interspecies transmission of SARS-CoV-2 between animals can also occur, as has been demonstrated from farmed mink to domestic cats [17]. Overall, the maintenance of SARS-CoV-2 within animal populations and the potential for zoonotic spillover emphasizes the need for further viral surveillance in animals.

The current gold standard method for the detection of SARS-CoV-2 in humans is real-time reverse-transcription polymerase chain reaction (rRT-PCR) [18]. Similarly, rRT-PCR has been used for the detection of SARS-CoV-2 infection in numerous animal species utilizing specimens from the respiratory tract (nasal/nasopharyngeal swabs, oral/oropharyngeal swabs, and tracheal washes) and the gastrointestinal tract (feces and rectal swabs) [19,20,21]. Given that animals, especially zoologic and wildlife species, are not often amenable to sampling from the respiratory tract without sedation and/or anesthesia, feces represents a promising, noninvasive sample type for SARS-CoV-2 surveillance. In humans, the median duration of shedding of non-variant SARS-CoV-2 viral RNA was shown to be significantly longer in feces (22 days) than in respiratory samples (18 days) [22]. Similarly, prolonged fecal shedding of non-variant SARS-CoV-2 viral RNA has been demonstrated in the Malayan tiger (*Panthera tigris jacksoni*; 14 days), Amur tiger (*Panthera tigris altaica*; 24 days), and African lion (*Panthera leo krugeri*; 30+ days) [23]. Additionally, shedding of replication-competent virus from feces, as indicated by successful viral isolation, has been demonstrated for the Amur tiger and African lion [23]. Therefore, feces represent a viable and clinically relevant sample type for nucleic acid amplification of SARS-CoV-2 in animals.

Loop-mediated isothermal amplification (LAMP) is an alternative nucleic acid amplification method first described by Notomi and colleagues in 2000 that utilizes four primers, recognizing a total of six distinct sequences on the target DNA and a strand-displacing DNA polymerase (such as *Bst* DNA polymerase, derived from the thermophilic bacterium *Geobacillus stearothermophilus*) to produce isothermal amplification at a uniform temperature of 65 °C in one hour [24,25]. The addition of two loop primers that hybridize to the stem-loops generated during the reaction can further accelerate the amplification such that the time to positivity is reduced to 30 min [26]. During the COVID-19 pandemic, reverse transcription loop-mediated isothermal amplification (RT-LAMP), which introduces an additional reverse transcriptase for the generation of complementary DNA (cDNA) from viral RNA, emerged as the preeminent isothermal amplification technique and rRT-PCR alternative for SARS-CoV-2 nucleic acid detection [25,27]. In particular, pH-based, colorimetric RT-LAMP may be more suitable for point-of-care detection of SARS-CoV-2 than rRT-PCR given that sample positivity is detected through a simple color change at the endpoint of the assay, owing to the release of protons during the incorporation of deoxynucleoside triphosphate (dNTP) by the strand-displacing DNA polymerase, with the increased acidity of the reaction detectable by pH indicator dyes such as phenol red [25]. Additionally, given that amplification occurs under isothermal conditions, colorimetric RT-LAMP can be performed utilizing a simple heat block or incubator, without the need for the sophisticated and expensive equipment (thermal cycler) required for rRT-PCR. In a systematic review and meta-analysis comparing the screening value of RT-LAMP and rRT-PCR for SARS-CoV-2 detection from human samples, rRT-PCR performed only slightly better than RT-LAMP in terms of sensitivity (96% vs. 92%) and specificity (100% vs. 99%) [18]. Given that RT-LAMP is suitable for regular testing at the point of care with only a marginal decrease in sensitivity and specificity compared to rRT-PCR, its utility may lie in the earlier detection of individuals with high viral loads; as such, RT-LAMP has been successfully deployed as a surveillance test for the detection of SARS-CoV-2 in human samples [28].

Though LAMP has been used extensively in veterinary medicine to detect other DNA and RNA viral pathogens [29], RT-LAMP has not been validated previously, to our knowledge, for SARS-CoV-2 detection in animals. Therefore, our study’s objective was to validate a commercial, pH-based, colorimetric RT-LAMP assay for the detection of SARS-CoV-2 in animal feces through the determination of the analytical sensitivity and specificity; the assay robustness under varying experimental conditions, including differing incubation lengths, incubation temperatures, and reaction volumes; the diagnostic sensitivity and specificity utilizing clinical fecal samples from a variety of animal species with comparison to rRT-PCR; and the reproducibility of the results through blinded method testing (BMT).

## 2. Materials and Methods

### 2.1. Viral Isolates

The analytical sensitivity, analytical specificity, and assay robustness were determined utilizing quantified Gamma-Irradiated SARS-Related Coronavirus 2 Isolate hCoV-19/USA/GA-EHC-2811C/2021 (Lineage B.1.1.529; Omicron Variant; BEI resources catalog #NR-56496). For blinded method testing, heat-inactivated SARS-Related Coronavirus 2, Isolate hCoV-19/USA/MD-HP05285/2021 (Lineage B.1.617.2; BEI resources catalog #NR-56128) was the Delta variant tested, and heat-inactivated SARS-Related Coronavirus 2 Isolate hCoV-19/USA/GA-EHC-2811C/2021 (Lineage B.1.1.529; BEI resources catalog #NR-56495) was the Omicron variant tested.

### 2.2. Fecal Suspension Preparation and Nucleic Acid Extraction

Fecal suspensions were prepared by swabbing fecal samples in four different locations using a cotton-tipped wooden applicator, then stirring or twirling the swab in an Eppendorf tube containing 1 mL of phosphate-buffered saline (PBS, pH 7.4). The swab was then removed while squeezing the tube to avoid loss of the suspension solution. Additional PBS was added such that the total volume of fecal suspension solution was 1 mL. The tube was then vortexed for 15 s and centrifuged for 2 min at 8000 rpm.

Nucleic acid extraction was performed using the MagMAX™ Pathogen RNA/DNA Kit on a KingFisher Flex machine (ThermoFisher, Waltham, MA, USA) utilizing 200 µL of fecal suspension solution for each sample. Extraction was performed according to the manufacturer’s instructions, except 400 µL of lysis/binding solution and 60 µL of elution buffer were utilized for each sample.

### 2.3. RT-LAMP Testing

RT-LAMP was performed using the SARS-CoV-2 Rapid Colorimetric LAMP Assay Kit (New England Biolabs, Ipswich, MA, USA), utilizing 12.5 μL of WarmStart^®^ Colorimetric LAMP 2X Master Mix with UDG, 2.5 μL of SARS-CoV-2 LAMP Primer Mix (N/E), 2.5 μL of guanidine hydrochloride, and 5.5 μL of nuclease-free water (collectively termed RT-LAMP Master Mix and totaling 23.0 μL), with 2.0 μL of extracted RNA (for a grand total of 25.0 μL per sample). The sequences for the N/E primer set are provided in Table A1. The negative (no template) control (NTC) was prepared utilizing the same volume of RT-LAMP Master Mix with an additional 2.0 μL of nuclease-free water. The positive control was prepared utilizing the same volume of RT-LAMP Master Mix with an additional 2.0 μL of SARS-CoV-2 Positive Control (N gene). RT-LAMP Master Mix was prepared and combined in a separate room using different laboratory coats to limit cross-contamination from previously amplified products. Unless otherwise specified (as in testing for assay robustness), RT-LAMP was performed according to the manufacturer’s instructions, with an incubation temperature of 65 °C and an incubation time of 30 min, using a Bio-Rad CFX Opus 96 Real-Time PCR instrument (Bio-Rad Laboratories, Hercules, CA, USA). At the conclusion of the assay, samples were placed onto white printer paper and photographed using a smartphone camera. Samples exhibiting a color change from pink to yellow were considered positive. Samples remaining pink were considered negative. Care was taken at the conclusion of each assay to discard reaction tubes without opening the lids, thereby minimizing cross-contamination.

### 2.4. rRT-PCR Testing

rRT-PCR was performed using the AgPath-ID™ One-Step RT-PCR kit (Thermo Fisher Scientific, Waltham, MA, USA), utilizing 12.5 μL of 2X reaction buffer, 1.0 μL of Xeno™, 1.0 μL of LIZ assay, 1.0 μL of 25X RT-PCR enzyme mix, 5.5 μL of biological-grade water, and 2.0 μL of CDC N1 primer/probe mix for each sample for the rRT-PCR Master Mix, which totaled 23.0 μL [30]. For each sample, 2.0 μL of extracted RNA was added to match the amount of template utilized in the RT-LAMP assay, for a grand total of 25.0 μL. rRT-PCR was performed for 40 amplification cycles on a Bio-Rad CFX Opus 96 Real-Time PCR instrument using Bio-Rad CFX Maestro software 2.3 (Bio-Rad Laboratories, Hercules, CA, USA). Cycle threshold (Ct) values were recorded for each sample. Samples with rRT-PCR Ct values of zero were considered negative, whereas samples with rRT-PCR Ct values ≤ 40 were considered positive.

### 2.5. Analytical Sensitivity

The analytical sensitivity was determined utilizing quantified SARS-CoV-2 GA-EHC-2811C/2021, which was diluted 10-fold at higher concentrations (~6.4 × 10^5^ to 941 genome copies), diluted 2-fold at lower concentrations (586, 282, 113, 72, 36, 19, 4, and 3 genome copies), and spiked into fecal suspensions prepared using SARS-CoV-2 negative feces from Felidae. The tube was then vortexed for 15 s and centrifuged for 2 min at 8000 rpm. The limit of detection (LoD) was defined according to the FDA Template for Developers of Molecular Diagnostic Tests as the lowest concentration at which at least 19 of 20 replicates were RT-LAMP positive [31].

### 2.6. Analytical Specificity

The analytical specificity (cross-reactivity) was determined through RT-LAMP wet testing of isolates or banked clinical samples known to be positive for other coronaviruses and sourced from humans, pigs, domestic cats, cattle, and bottlenose dolphins (Table 1). Notably, certain samples were known to be positive for multiple viruses, including one pig isolate that was positive for PEDV, PDCoV, and TGEV and one bovine isolate that was positive for BCoV and BRSV (a pneumovirus). The simulated SARS-CoV-2 positive sample was created by spiking quantified SARS-CoV-2 GA-EHC-2811C/2021 into a fecal suspension prepared using SARS-CoV-2 negative feces from Felidae.

### 2.7. Assay Robustness

Assay robustness was assessed by performing RT-LAMP under potential stress conditions, as recommended by the FDA [31], including incubation lengths, incubation temperatures, and reaction volumes that differ from those recommended by the manufacturer. For assessment of differing incubation lengths, quantified SARS-CoV-2 GA-EHC-2811C/2021 was diluted to 1.0 × 10^6^, 1.0 × 10^5^, 4024, 236, 2, and 0 genome copies and tested with RT-LAMP at incubation lengths ranging from 0 min to 60 min with increments of 15 min. For the assessment of differing reaction volumes, the same viral dilutions were tested by adding variable amounts of RT-LAMP Master Mix to reach final reaction volumes of 25.0 μL (23.0 μL + 2.0 μL of template), 20.0 μL (18.0 μL + 2.0 μL of template), and 10.0 μL (8.0 μL + 2.0 μL of template). For the assessment of differing incubation temperatures, two viral dilutions (~1000 and ~100 genome copies) were tested with RT-LAMP at incubation temperatures ranging from 60 °C to 70 °C with increments of 1 °C. One NTC sample was included for each experimental manipulation.

### 2.8. Banked Clinical Fecal Samples

Permission was obtained from the submitting zoological institutions to repurpose fecal specimens previously submitted to the University of Illinois Veterinary Diagnostic Laboratory for SARS-CoV-2 rRT-PCR testing for this study. Feces were collected noninvasively by the submitting institutions. Fecal samples were stored at −80 °C between initial rRT-PCR testing and use within this study.

In total, 126 fecal samples from 28 unique animals belonging to 7 different animal species and 3 different tiger subspecies were tested (Table 2), including the Sumatran tiger (*Panthera tigris sumatrae*), Amur tiger (*Panthera tigris altaica*), Malayan tiger (*Panthera tigris jacksoni*), African lion (*Panthera leo*), cheetah (*Acinonyx jubatus*), Asian small-clawed otter (*Aonyx cinereus*), white-cheeked gibbon (*Nomascus leucogenys*), bearded emperor tamarin (*Saguinus imperator subgrisescens*), and giant anteater (*Myrmecophaga tridactyla*). The total number of unique animals sampled differs from the total number of fecal samples since feces were collected from certain animals at multiple timepoints over the course of a SARS-CoV-2 outbreak or pooled in duplicate or triplicate from multiple animals of the same species at a single timepoint. All fecal samples previously determined to be SARS-CoV-2 positive were from *Panthera*. All fecal samples previously determined to be SARS-CoV-2 negative were from *Panthera* and the remaining species (cheetah, Asian small-clawed otter, white-cheeked gibbon, bearded emperor tamarin, and giant anteater).

### 2.9. Diagnostic Sensitivity and Specificity

The diagnostic sensitivity of RT-LAMP was determined for all rRT-PCR positive clinical fecal samples irrespective of the Ct value (variable viral load), for clinical fecal samples with rRT-PCR Ct values ≤ 33 (moderate-to-high viral load), and for clinical fecal samples with rRT-PCR Ct values ≤ 25 (high viral load), similar to the cut-offs utilized by Fowler and colleagues [32]. The diagnostic sensitivity was defined as the percentage of rRT-PCR positive samples within the specified Ct interval that were also positive when evaluated with the RT-LAMP assay. The diagnostic specificity of RT-LAMP was determined for all rRT-PCR negative (Ct value = 0) clinical fecal samples. The diagnostic specificity was defined as the percentage of rRT-PCR negative samples that were simultaneously negative when evaluated with the RT-LAMP assay. For both the diagnostic sensitivity and specificity, Wilson’s method for binomial confidence intervals was used to construct 95% confidence intervals using the R package binom, as utilized previously by Dao Thi and colleagues [33,34].

### 2.10. Blinded Method Testing

Blinded method testing (BMT) is an exercise in which an independent laboratory prepares and ships samples to the originating laboratory for analysis, ensuring that testing is conducted in a manner that minimizes unconscious bias. In this study, BMT samples were prepared by the laboratory in the FDA’s Veterinary Laboratory Investigation and Response Network (Vet-LIRN) [35] and included two SARS-CoV-2 viral variants of concern (Delta and Omicron), each prepared in two different sample matrices (feces from Felidae and PBS). The University of Illinois Veterinary Diagnostic Laboratory, which conducted the testing, was blinded to the identity of the samples, including the concentration of virus spiked into each sample, the number of replicates for each concentration, and whether samples were positive or negative. A total of 120 unknown test samples (500 µL each) were prepared by Vet-LIRN and shipped for analysis. Each sample was prepared by spiking 450 µL of fecal suspension solution or PBS with 50 µL of serially diluted virus (either Delta or Omicron; positive samples) or PBS (negative samples). The fecal suspension solution was prepared by wiping five cotton swabs into semi-solid feces and placing the swabs into 5 mL of PBS. To estimate the amount of semi-solid organic matter (feces) present in the final suspension, swabs were gravimetrically weighed before and after wiping into feces [36]. The fecal suspension solution in this BMT was estimated to contain 13.8% semi-solid feces (organic matter by weight) in PBS. For each sample matrix and viral variant, 20 samples were tested with RT-LAMP and rRT-PCR, including 4 negative samples and 16 positive samples varying in target concentration from 250 to 2500 genome copies per reaction, for a total of 80 samples. An additional 40 samples were prepared and archived for possible follow-up analysis if needed. Samples were analyzed over the course of two days.

## 3. Results

### 3.1. Analytical Sensitivity of RT-LAMP

**Figure 1:**
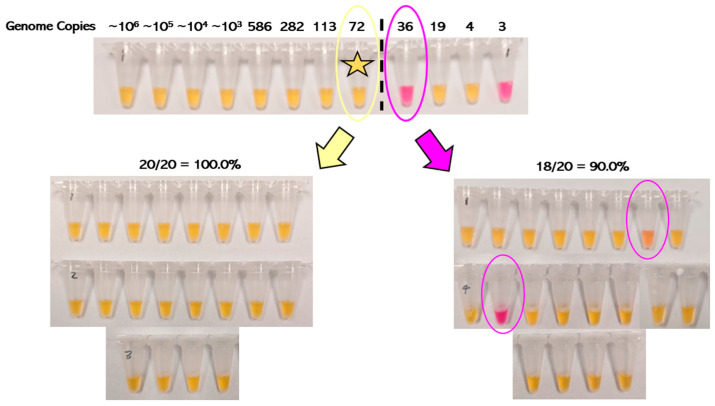
Determination of the limit of detection of simulated positive animal fecal samples spiked with the Omicron variant of concern. The limit of detection (LoD) was defined according to FDA guidelines as the lowest concentration at which at least 19 of 20 replicates were RT-LAMP positive and was determined to be 72 viral copies in this case, with stochastic detection of SARS-CoV-2 identified at lower genome copy levels. Positive samples turned yellow, whereas negative samples remained pink. Of note, one of the two replicates deemed negative at a genome copy level of 36 (below the LoD) had an ambiguous color change.

The RT-LAMP results show that SARS-CoV-2 could be detected in simulated positive animal fecal samples spiked with as low as 72 genome copies and up to ~6.4 × 10^5^ genome copies of the Omicron variant (Figure 1). At a level of 72 genome copies per reaction, all 20 replicates (100.0%) were positive, whereas at a level of 36 genome copies per reaction, only 18 out of 20 replicates (90.0%) were positive. Therefore, the LoD was determined to be 72 genome copies for the RT-LAMP assay. Interestingly, there was stochastic detection of SARS-CoV-2 even at 10-fold lower concentrations (as low as four genome copies), which has been reported previously by Amaral and colleagues [37]. Additionally, positive samples with concentrations below the limit of detection occasionally demonstrated ambiguous or intermediate color changes, which is a known drawback of the pH indicator dye phenol red when used in colorimetric LAMP [25].

### 3.2. Analytical Specificity of RT-LAMP

**Figure 2:**
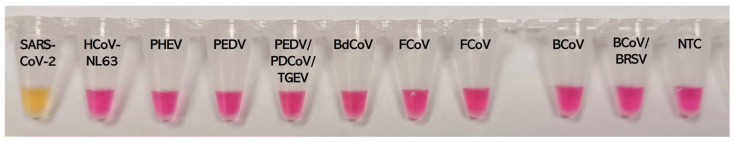
Determination of the analytical specificity of the RT-LAMP assay for SARS-CoV-2. The RT-LAMP assay was highly specific for SARS-CoV-2 and did not cross-react with other human and animal coronavirus isolates or positive clinical samples, including (from left to right) the human coronavirus NL63 (HCoV-NL63) isolate, porcine hemagglutinating encephalomyelitis virus (PHEV) isolate, porcine epidemic diarrhea virus (PEDV)-positive feces, PEDV/porcine deltacoronavirus (PDCoV)/transmissible gastroenteritis virus (TGEV) isolate, bottlenose dolphin coronavirus (BdCoV)-positive feces, feline coronavirus (FCoV)-positive lung, FCoV-positive pooled liver and kidney, bovine coronavirus (BCoV)-positive feces, and BCoV/bovine respiratory syncytial virus (BRSV) isolate. The no-template control (NTC) remained negative, as expected. Positive samples turned yellow, whereas negative samples remained pink.

Wet testing indicated that the RT-LAMP assay was highly specific for SARS-CoV-2 and did not detect other human (HCoV-NL63), porcine (PHEV, PEDV, PDCoV, and TGEV), bovine (BCoV), feline (FCoV), or bottlenose dolphin (BdCoV) coronaviruses (Figure 2).

### 3.3. Robustness of RT-LAMP

#### 3.3.1. Incubation Temperature

**Figure 3:**
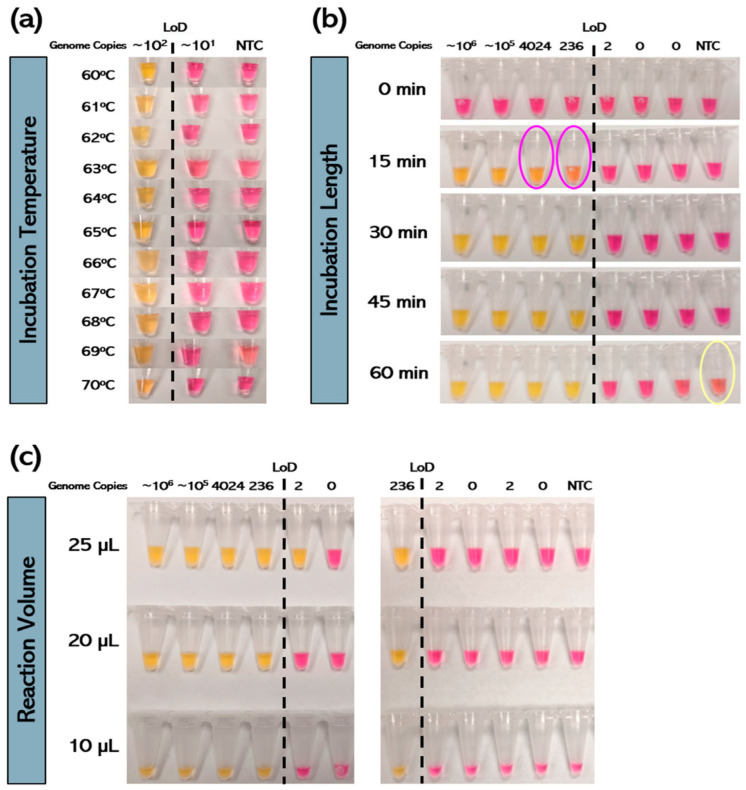
Assessment of the robustness of the RT-LAMP assay under various stress conditions. The limit of detection (LoD) is shown as a dotted line. Positive samples turned yellow, whereas negative samples remained pink. (**a**) RT-LAMP is valid at incubation temperatures ranging from 60 °C to 70 °C. (**b**) RT-LAMP is valid at incubation times ranging from 30 to 45 min. However, at 15 min, there is an ambiguous or intermediate color change in expected positives with genome copy levels near the LoD, and at 60 min, there is an ambiguous or intermediate color change in the NTC, which should remain negative. (**c**) RT-LAMP is valid at reaction volumes of 10 µL to 25 µL.

The expected results were obtained with RT-LAMP for isothermal amplification temperatures ranging from 60 °C to 70 °C, meaning differences of 5 °C in either direction from the manufacturer’s recommended incubation temperature of 65 °C appear well tolerated (Figure 3a).

#### 3.3.2. Incubation Length

The expected results were obtained with RT-LAMP for isothermal amplification lengths of 30 min and 45 min (Figure 3b). These findings match well with the manufacturer’s recommendations for this assay, which are to incubate samples for 30 min, with possible extension to 40 min to maximize sensitivity in samples expected to have very low copy levels [38]. With an incubation time of 15 min, there was ambiguous or intermediate color change in samples just above the LoD, suggesting that shorter incubation times lead to increased false negatives. With incubation times of 60 min, there was an ambiguous or intermediate color change in the NTC, suggesting that longer incubation times lead to increased false positives. Autoamplification of primer dimers within the NTC has been shown to occur with RT-LAMP following prolonged incubation times of ≥ 40 to 60 min and could explain these results [25].

#### 3.3.3. Reaction Volume

The expected results were obtained with RT-LAMP for samples containing identical amounts of template (2.0 μL) and reaction volumes of 25.0 μL (23.0 μL of RT-LAMP Master Mix), 20.0 μL (18.0 μL of Master Mix), and 10.0 μL (8.0 μL of Master Mix), meaning reductions in the RT-LAMP Master Mix of up to 15.0 μL appear to be well tolerated (Figure 3c).

### 3.4. Diagnostic Sensitivity and Specificity of RT-LAMP

**Figure 4:**
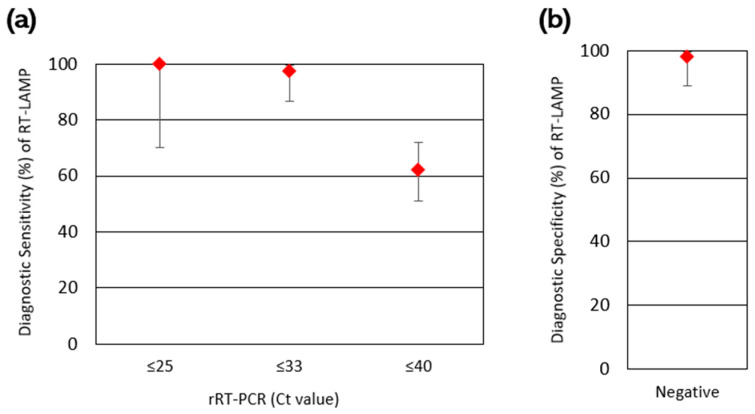
Comparison of RT-LAMP to rRT-PCR for the detection of SARS-CoV-2 RNA in clinical fecal samples from animals. (**a**) The diagnostic sensitivity of RT-LAMP, with stratification of positive samples based on their rRT-PCR Ct values into high viral load (Ct ≤ 25), moderate to high viral load (Ct ≤ 33), and variable viral load (all positive samples, with positivity defined as Ct ≤ 40) groupings. The error bars indicate the corresponding Wilson’s binomial 95% confidence intervals. (**b**) The diagnostic specificity of RT-LAMP, with the error bars indicating the corresponding Wilson’s 95% confidence interval.

Of 126 total clinical fecal samples tested, 79 were rRT-PCR positive and 47 were rRT-PCR negative (Table 3). Of these rRT-PCR positive samples, 49 were RT-LAMP positive and 30 were RT-LAMP negative, for an overall diagnostic sensitivity of 62.0% (Wilson’s 95% confidence interval (CI): 51.0% to 71.9%). However, when only considering rRT-PCR positive samples with Ct values ≤ 33 (moderate-to-high viral load samples), the diagnostic sensitivity was 97.4% (95% CI: 86.8% to 99.5%), with only 1 sample with a Ct value of 32.7 not detected by RT-LAMP out of 39 total samples. The remaining 38 samples with Ct values of ≤32.4 were all detected. Similarly, when only considering rRT-PCR positive samples with Ct values ≤ 25 (high viral load), the diagnostic sensitivity was even higher at 100.0% (95% CI: 70.1% to 100.0%), with all nine samples detected with RT-LAMP. Of the 47 rRT-PCR negative samples, 46 were RT-LAMP negative and 1 was RT-LAMP positive, for an overall diagnostic specificity of 97.9% (95% CI: 88.9% to 99.6%). Given that this RT-LAMP positive sample was previously rRT-PCR positive with a Ct value of 37.6 during initial testing utilizing 5.0 μL (rather than 2.0 μL) of template, RT-LAMP positivity could potentially be explained by the stochastic detection of SARS-CoV-2 by RT-LAMP at lower concentrations, though false positivity due to cross-contamination or primer–primer interactions cannot be ruled out. The diagnostic sensitivity and diagnostic specificity results are summarized in Figure 4.

**Figure 5:**
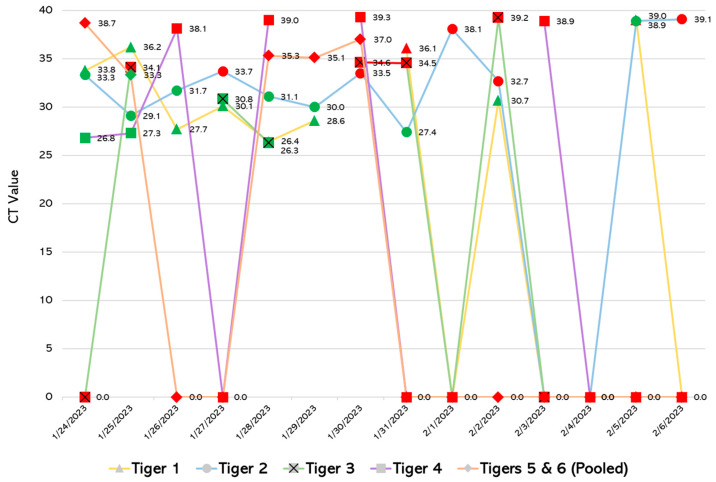
SARS-CoV-2 outbreak in Sumatran tigers (*Panthera tigris sumatrae*) at a zoological institution, demonstrating the variable rRT-PCR Ct values of clinical fecal samples over time. For each clinical fecal sample and timepoint, a green marker indicates the detection of SARS-CoV-2 RNA using RT-LAMP, whereas a red marker indicates a lack of detection of SARS-CoV-2 RNA using RT-LAMP.

The clinical relevance of the decreased sensitivity of RT-LAMP compared to rRT-PCR may be best interpreted in the context of a SARS-CoV-2 outbreak. When RT-LAMP was retrospectively applied to an outbreak of SARS-CoV-2 (Omicron variant) in six Sumatran tigers (*Panthera tigris sumatrae*) at a zoological institution, there was reliable detection of SARS-CoV-2 RNA in positive fecal samples with Ct values <32.7, with stochastic detection of SARS-CoV-2 RNA in positive fecal samples with Ct values as high as 38.9 (Figure 5). SARS-CoV-2 viral RNA shedding in feces was prolonged and highly variable, with intermittent periods of nondetectable shedding. These fecal shedding dynamics are similar to those previously identified by Bartlett and colleagues for tigers and lions infected with non-variant SARS-CoV-2 [23]. Importantly, RT-LAMP could have detected SARS-CoV-2 RNA in every tiger (or tiger pairing for pooled samples) at least once during the outbreak. Additionally, if run in-house, RT-LAMP could have allowed for the earlier detection of preliminary positive animals prior to send-out confirmatory rRT-PCR testing.

### 3.5. Blinded Method Testing of RT-LAMP

**Figure 6:**
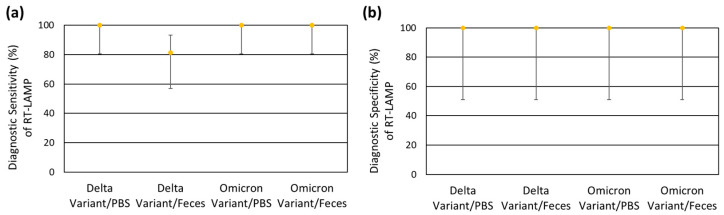
Summary of the performance of RT-LAMP for the detection of SARS-CoV-2 RNA during BMT, with stratification of simulated samples based on the viral variant (Delta or Omicron) and sample matrix (PBS or feces). (**a**) The diagnostic sensitivity of RT-LAMP, with error bars indicating the corresponding Wilson’s binomial 95% confidence intervals. (**b**) The diagnostic specificity of RT-LAMP, with error bars indicating the corresponding Wilson’s binomial 95% confidence intervals.

The results of blinded method testing are summarized in Table 4. The diagnostic sensitivity for the Omicron variant was 100.0% (Wilson’s 95% CI: 80.6% to 100.0%) regardless of the sample matrix type (PBS or feces). The diagnostic sensitivity for the Delta variant was similarly 100.0% (Wilson’s 95% CI: 80.6% to 100.0%) for samples in the PBS matrix. However, the diagnostic sensitivity for the Omicron variant was 81.3% (Wilson’s 95% CI: 57.0% to 93.4%) for samples in the fecal matrix, with only 13 out of 16 positive samples detected with RT-LAMP. The three undetected samples had an intended concentration of 250 genome copies per reaction, though the average rRT-PCR Ct value of these samples was 33.7 when analyzed in-house. These results suggest that the RT-LAMP assay may be slightly more sensitive for the detection of SARS-CoV-2 viral RNA in fecal samples of animals infected with the Omicron variant compared to the Delta variant. Given that the Delta variant was detected at this intended concentration level when viral RNA was spiked into PBS, a matrix effect from feces may be contributing to the slightly decreased sensitivity. The diagnostic specificity was 100.0% (Wilson’s 95% CI: 51.0% to 100.0%) for all experiment manipulations and did not differ between the evaluated viral variants (Delta and Omicron) and sample matrices (PBS and feces). The diagnostic sensitivity and diagnostic specificity results are summarized in Figure 6.

## 4. Discussion

We validated a sensitive and specific RT-LAMP assay for detecting SARS-CoV-2 viral RNA in animal feces. The diagnostic specificity of the RT-LAMP assay (97.9%) for the detection of SARS-CoV-2 in clinical fecal samples from animals was comparable to rRT-PCR. Overall, RT-LAMP was less sensitive than rRT-PCR, with a diagnostic sensitivity of 62.0%, meaning SARS-CoV-2-positive animals with low viral loads could potentially be missed by the RT-LAMP assay. However, RT-LAMP was highly sensitive for detecting SARS-CoV-2 viral RNA in moderate to high (Ct value ≤ 33) and high viral load (Ct value ≤ 25) samples, with diagnostic sensitivity values of 97.4% and 100.0%, respectively. These findings are comparable to the diagnostic sensitivities reported for RT-LAMP assays validated for human samples, with reliable detection of SARS-CoV-2 in samples with corresponding rRT-PCR Ct values < 30 [39]. One potential limitation of our study is the use of rRT-PCR with a single gene target as the gold standard comparator assay for RT-LAMP. The overall sensitivity of rRT-PCR with multiple gene targets is slightly greater than rRT-PCR with a single gene target, meaning the comparison of RT-LAMP to rRT-PCR with a single gene target may have slightly exaggerated the sensitivity of RT-LAMP [18]. Another limitation of our study was that *Panthera* were overrepresented, contributing 109 out of 126 (86.5%) of the clinical fecal samples utilized in our study, with tigers (*Panthera tigris*) contributing 94 out of 126 (74.6%) of the clinical samples evaluated. However, *Panthera* were also overrepresented in terms of overall confirmed cases in non-domestic species submitted to veterinary diagnostic laboratories. Fecal matrices vary between species, with higher microbial diversity identified for herbivores as compared to carnivores such as *Panthera*, suggesting that subsequent RT-LAMP validation for fecal samples from herbivorous species may be warranted [40]. More broadly, validation across a wider range of SARS-CoV-2 susceptible taxa, including herbivores (cervids), domestic carnivores (dogs, cats, and mustelids), rodents, and non-human primates, is warranted.

The relationship between SARS-CoV-2 RNA detection, viral load, and infectivity is not completely understood, though the present data suggest that, in humans, Ct values ≥34 are unlikely to be infectious [41,42]. When extrapolating this infectivity cut off to animals, the decreased sensitivity of RT-LAMP for detecting SARS-CoV-2 RNA in samples with Ct values ≥ 34 becomes much less clinically meaningful. Furthermore, the existing epidemiologic data on SARS-CoV-2 in humans suggests that test accessibility, frequency, and speed of reporting are more important than test sensitivity for SARS-CoV-2 outbreak prevention [43,44,45]. When applied retrospectively to an outbreak of SARS-CoV-2 in Sumatran tigers at a zoological institution, RT-LAMP was able to detect SARS-CoV-2 RNA in at least one fecal sample from every tiger (or tiger pairing) during the course of the outbreak. Thus, we can conclude that in an animal outbreak of SARS-CoV-2, RT-LAMP surveillance in a point-of-care setting (such as a veterinary clinic or zoo) would likely allow for the earlier detection of even presymptomatic or subclinical animals with moderate to high or high viral loads that are likely to be infectious, thereby allowing for earlier implementation of quarantine and control efforts to limit viral spread.

BMT demonstrated that the RT-LAMP assay yielded reproducible results, even under the blinded conditions of the collaborative study. RT-LAMP showed slightly better sensitivity for detecting SARS-CoV-2 RNA in simulated clinical fecal samples spiked with the Omicron variant compared to the Delta variant. This difference was only observed at the lowest tested target concentration (250 copies per reaction), corresponding to an average Ct value of 33.7 for the Delta variant and 34.8 for the Omicron variant. At this low concentration, only three out of six (50.0%) Delta variant samples were detected, while all six (100.0%) Omicron variant samples were detected. Interestingly, sensitivity differences were noted when viral RNA was spiked into PBS rather than feces, suggesting some fecal matrix effect. This finding makes sense when we consider the numerous inhibitors present in fecal matter, including proteins (hemoglobin and immunoglobulins), pigments (bilirubin), polysaccharides (glycogen), fats (triglycerides), and acids (bile acids and phytic acid), and the abundance of competing nucleic acids from the intestinal microbiota, all of which may have interfered with SARS-CoV-2 RNA detection [46,47,48]. Importantly, the fecal suspension used in this BMT study was estimated to contain 13.8% semi-solid feces (organic matter by weight) in PBS. Unfortunately, the organic matter concentration in fecal test samples is often not reported by other studies, making meaningful comparisons of assay sensitivities across different matrices and studies challenging. Standardized reporting of fecal sample composition would improve assay evaluation and facilitate more accurate inter-study and inter-matrix comparisons.

The impact of mutations on primer binding cannot be ruled out as a potential contributor to the decreased sensitivity of RT-LAMP for the detection of the Delta variant as compared to the Omicron variant in animal fecal samples. The first primer set utilized in the RT-LAMP assay detects the N2 region within the N gene, encoding the structural N (nucleocapsid) protein, which contributes to viral assembly and budding, whereas the second primer set detects the E1 region within the E gene, encoding the structural E (envelope) protein, which is a hydropathic membrane protein containing numerous valine and leucine residues [49]. In general, the N gene and the E gene of SARS-CoV-2 are highly conserved, with fewer mutations than the spike glycoprotein [49]. However, mutations in the N protein of SARS-CoV-2 have been identified, with R203K and G204R being the most common [50]. Similarly, differences in the amino acid composition of the E protein of SARS-CoV-2, particularly of the C-terminal domain, have been identified, such as S55F, V62F, and R69I [51].

Variants of concern are often named based on alterations in transmissibility or virulence, especially due to mutations in the spike glycoprotein, rather than sequence variations that impact primer binding and resultant nucleic acid detection, making the performance of the RT-LAMP assay difficult to predict solely based on the SARS-CoV-2 variant of concern in question [39]. However, the New England Biolabs Primer Monitor Tool, which compares the sequences of primers with that of SARS-CoV-2 variants deposited in GenBank, makes it easier to predict mutations that might impact primer binding and the detection of SARS-CoV-2 using RT-LAMP [52]. Sequence comparison between the specific Delta and Omicron variant viruses utilized during blinded method testing and the RT-LAMP primers revealed no mismatches for the Delta variant and one mismatch for the Omicron variant in the forward inner primer for the envelope gene (E1-FIP), suggesting that primer binding is unlikely to have played a role in the decreased sensitivity of RT-LAMP for the detection of the Delta variant as compared to the Omicron variant in this case. Single point mutations can have profound effects on the performance of rRT-PCR for SARS-CoV-2 detection, decreasing sensitivity by ten to one-hundred-fold and by up to 7.6 Ct values depending on the mutation and its position [53]. In contrast, single point mutations at most positions within an RT-LAMP primer set still result in the detection of SARS-CoV-2, with only a marginal reduction in the amplification speed of the assay [53]. Therefore, even SARS-CoV-2 RNA mutations that arise within primer binding sites seem well tolerated and may not necessarily impact the performance of RT-LAMP or necessitate the development of new primers. Even still, subsequent RT-LAMP validation for emerging variants may be warranted. In addition to the relatively high inclusivity for viral variants, the RT-LAMP N/E primer set demonstrates high exclusivity for SARS-CoV-2, as evidenced by the lack of detection of other tested human (HCoV-NL63), porcine (PHEV, PEDV, PDCoV, and TGEV), bovine (BCoV), feline (FCoV), or bottlenose dolphin (BdCoV) coronaviruses.

RT-LAMP testing under variable stress conditions revealed the overall robustness of the assay. The detection of SARS-CoV-2 RNA near the limit of detection of the assay (72 genome copies) did not differ for incubation temperatures of 60 °C to 70 °C, incubation lengths of 30 min to 45 min, and total reaction volumes of 25.0 μL, 20.0 μL, and 10.0 μL. The two enzymes utilized in the RT-LAMP assay include a strand-displacing DNA polymerase (New England Biolabs *Bst* 2.0 WarmStart^®^ DNA Polymerase), which has a manufacturer-reported optimal reaction performance of 60 °C to 72 °C, and a reverse transcriptase (New England Biolabs WarmStart^®^ RTx Reverse Transcriptase), which has a reported optimal reaction performance of 55 °C to 65 °C, with the overlap in temperature range (65 °C) representing the optimum incubation temperature for the RT-LAMP assay. The reproducibility of RT-LAMP results at tested incubation temperatures of 60 °C to 70 °C suggests that suboptimal performance by the reverse transcriptase at temperatures of 66 °C to 70 °C may be relatively well tolerated by the assay. The reproducibility of RT-LAMP results for incubation lengths of 30 min and 45 min are in line with the manufacturer’s recommendations to incubate for 30 min, with possible extension to 40 min for maximal sensitivity in samples with low genome copy levels. Shorter incubation times of 15 min led to ambiguous or intermediate color change for samples just above the LoD, likely due to insufficient amplification and thus insufficient acidification for a complete color change of phenol red from pink (pH~8.0) to yellow (pH~6.0) [54]. Longer incubation times of 60 min led to ambiguous or intermediate color change in the NTC, likely due to the autoamplification of primer dimers [25]. The reproducibility of RT-LAMP results for the manufacturer-recommended total reaction volume of 25 μL and the other tested reaction volumes of 20 μL and 10 μL suggests that sufficient amplification of SARS-CoV-2 RNA can still occur in spite of reduced RT-LAMP Master Mix, which contains weakly basic Tris reaction buffer, phenol red, RT-LAMP enzymes and primers, the contamination reduction components deoxyuridine triphosphate (dUTP) and uracil DNA glycosylase (UDG), the reaction speed and sensitivity enhancer guanidine hydrochloride, and nuclease-free water [25,55]. Altogether, these results suggest that the RT-LAMP assay is robust to slight changes in the incubation temperature, incubation length, and reaction volume that might result from operator error or improperly calibrated laboratory equipment, though the manufacturer’s recommendations to incubate samples prepared at a total reaction volume of 25 μL for 30 min at 65 °C should still be followed whenever possible.

The development of a true point-of-care assay for the detection of SARS-CoV-2 RNA in animal feces will require simplification of the assay and associated machinery. Our validation utilized a real-time thermal cycler to carry out RT-LAMP experiments rather than a more accessible and low-cost heat block or incubator that would allow for isothermal amplification to be carried out in more resource-limited settings. Therefore, subsequent validation with a heat block or incubator is warranted before the RT-LAMP assay could be deployed in a point-of-care setting. Additionally, our protocol necessitates nucleic acid extraction of fecal suspension solutions prior to RT-LAMP analysis, which may be impractical in a point-of-care setting. Extraction-free RT-LAMP has been performed successfully for SARS-CoV-2 RNA detection in human respiratory samples (nasal and nasopharyngeal swabs) [28]. Despite the variability in pH and viscosity and the presence of inhibitors in human saliva, extraction-free, pH-based, colorimetric LAMP has also been utilized with this sample type [56], suggesting that extraction-free RT-LAMP might be feasible for other complex sample types such as feces. Successful extraction-free, fluorescence-based RT-LAMP has been reported for SARS-CoV-2 detection in raw sewage [57]; however, attempts at subsequent extraction-free, colorimetric RT-LAMP for SARS-CoV-2 RNA detection utilizing wastewater samples were unsuccessful, with prior extraction required to reduce matrix inhibition [58]. Similarly, extraction-free, colorimetric, RT-LAMP was unsuccessful when deployed for the detection of *Scotophilus* bat-CoV 512 (an *Alphacoronavirus*) RNA in bat feces, necessitating prior extraction to reduce matrix inhibition [47]. Therefore, it may be the case that extraction-free, colorimetric RT-LAMP is invalid for the detection of SARS-CoV-2 RNA in complex sample matrices such as feces and sewage due to overwhelming inhibition. Extraction-free, fluorescent LAMP has been utilized for the detection of SARS-CoV-2 RNA and *Helicobacter pylori* (a bacterial pathogen) DNA in environmental waters and human feces, respectively, by employing a hydrogel to limit the diffusion of environmental inhibitors [59,60], suggesting that the implementation of novel methods to overcome matrix inhibition holds promise for future successful, extraction-free RT-LAMP detection of SARS-CoV-2 RNA in animal feces. Targeted dilution based on quantitative inhibitor analysis has shown promise for reducing inhibition and thus improving the detection of RNA viruses in environmental water samples using rRT-PCR [61]. With this method, the dilution factor is calculated based on the change in the quantification cycle of an RNA standard following spiking into a clinical sample [61]. Unfortunately, this necessitates an additional rRT-PCR step prior to dilution and testing of the clinical sample with RT-LAMP, thereby reducing the detection speed and requiring the use of a quantitative thermal cycler [61].

Given that pH-based, colorimetric methods depend critically on the initial starting pH of the reaction [25], which may be highly variable for complex matrices such as feces, fluorescent RT-LAMP may be more desirable for use with extraction-free protocols. Additionally, fluorescent RT-LAMP could allow for the assessment of inhibition through multiplexing utilizing internal amplification controls [61]. In resource-limited settings, fluorescent readouts can be achieved utilizing low-cost fluorometers and lightboxes [62,63]. Alternatively, pH-based, colorimetric RT-LAMP could be utilized in a point-of-care setting following more simplistic extraction and purification methods, utilizing glass milk or magnetic beads [64,65]. Paper-based biosensors integrating nucleic acid extraction, LAMP, and lateral flow detection with simple colorimetric readouts have been successfully deployed to detect bacteria, including *Escherichia coli* and *Streptococcus pneumoniae*, in human blood samples, with a sample-to-answer time of approximately one hour [66]. Handheld battery-powered heating devices, when coupled with biosensors, allow for isothermal amplification to be performed in remote settings [66]. Integrated cartridge designs utilizing isothermal nucleic acid amplification methods are a potential future direction of point-of-care testing for SARS-CoV-2, allowing for “sample-in-answer-out” detection in resource-limited settings [67]. The development of a point-of-care RT-LAMP protocol for the detection of SARS-CoV-2 in animal feces warrants further investigation, though our study establishes the clinical utility of the assay and lays the groundwork for future method refinement and comparison.

## 5. Conclusions

In conclusion, we successfully validated a commercial, pH-based, colorimetric RT-LAMP assay for the detection of SARS-CoV-2 RNA in animal feces. The RT-LAMP assay was slightly less sensitive than rRT-PCR and could reliably detect SARS-CoV-2 RNA in fecal samples from animals with moderate to high (Ct value ≤ 33) and high (Ct value ≤ 25) viral loads that were likely to be infectious. However, the assay could not reliably detect SARS-CoV-2 RNA in fecal samples with low viral loads (Ct value > 33). The assay was highly specific for SARS-CoV-2 and did not detect other human or animal coronaviruses. The assay was highly robust and valid at incubation lengths of 30 to 45 min, incubation temperatures of 60 to 70 °C, and reaction volumes of 10 to 25 µL. The reproducibility of the RT-LAMP assay was established through blinded method testing performed in conjunction with an outside laboratory. RT-LAMP could ultimately be deployed as a rapid, low-cost, noninvasive, point-of-care nucleic acid test for SARS-CoV-2 surveillance in veterinary clinics and zoos for earlier detection of SARS-CoV-2 infection and thus earlier implementation of control efforts and medical interventions to minimize viral spread and animal mortality.

## Figures and Tables

**Table 1:** Summary of human and animal viruses tested for evaluation of the analytical specificity of the RT-LAMP assay.

Viruses Tested	Animal Species	Type of Sample
Human coronavirus NL63 (HCoV-NL63)	Human	Isolate ^1^
Porcine hemagglutinating encephalomyelitis virus (PHEV)	Pig	Isolate
Porcine epidemic diarrhea virus (PEDV)	Pig	Feces
PEDV/porcine deltacoronavirus (PDCoV)/transmissible gastroenteritis virus (TGEV) ^2^	Pig	Isolate
Bottlenose dolphin coronavirus (BdCoV)	Bottlenose dolphin	Feces
Feline coronavirus (FCoV)	Domestic cat	Lung
FCoV	Domestic cat	Liver and Kidney
Bovine coronavirus (BCoV)	Bovine	Feces
BCoV/bovine respiratory syncytial virus (BRSV) ^2^	Bovine	Isolate ^3^
SARS-CoV-2	Felidae	Feces

^1^ The HCoV-NL63 isolate was obtained from BEI resources (catalog # NR-470). ^2^ These samples were prepared by mixing two or three viral isolates to be utilized as positive extraction controls during viral testing. ^3^ The BCoV/BRSV isolate was obtained from Ailam Lim at the Wisconsin Veterinary Diagnostic Laboratory.

**Table 2:** Summary of study population from which clinical fecal samples were derived for evaluation of the diagnostic sensitivity and specificity of the RT-LAMP assay.

Animal Species	Number of Fecal Samples	Number of Animals
Sumatran tiger	79	7
African lion	15	8
Amur tiger	8	3
Malayan tiger	7	2
Cheetah	7	1
Asian small-clawed otter	5	1
White-cheeked gibbon	3	3
Bearded emperor tamarin	1	2
Giant anteater	1	1
Total	126	28

**Table 3:** Summary of the results of rRT-PCR and RT-LAMP testing of 126 banked clinical fecal samples from animals stratified into Ct value bins, with the calculated diagnostic sensitivity (DSe) and diagnostic specificity (DSp). The DSe was defined as the percentage of rRT-PCR positive samples within the specified Ct interval that were also positive when evaluated with the RT-LAMP assay. The DSp was defined as the percentage of rRT-PCR negative samples that were simultaneously negative when evaluated with the RT-LAMP assay.

RT-LAMP							
		Ct	Positive	Negative	Total	DSe	DSp
rRT-PCR	Positive	≤25	9	0	9	100.0%	-
		≤33	38	1	39	97.4%	-
		≤40	49	30	79	62.0%	-
	Negative	Negative	1	46	47	-	97.9%
	Total		50	76	126		

**Table 4:** Summary of the results of BMT of 80 simulated clinical samples, with 20 samples (16 positive and 4 negative) tested for each viral variant and matrix combination, including (**A**) the Delta variant in PBS matrix, (**B**) the Delta variant in fecal matrix, (**C**) the Omicron variant in PBS matrix, and (**D**) the Omicron variant in fecal matrix. The target concentration reflects the intended number of genome copies per reaction for samples prepared by FDA Vet-LIRN.

**(A) Delta Variant/PBS Matrix**				**RT-LAMP**		
		**Target** **Concentration/** **Reaction**	**Average Ct**	**Positive**	**Negative**	**Total**
rRT-PCR	Positive	250	33.2	6	0	6
		500	31.9	2	0	2
		1000	31.1	6	0	6
		2500	29.7	2	0	2
	Negative	0	0.0	0	4	4
		Total		16	4	20
(B) Delta Variant/Fecal Matrix *				**RT-LAMP**		
		**Target** **Concentration/** **Reaction**	**Average Ct**	**Positive**	**Negative**	**Total**
rRT-PCR	Positive	250	33.7	3	3	6
		500	32.9	2	0	2
		1000	32.4	6	0	6
		2500	30.4	2	0	2
	Negative	0	0.0	0	4	4
		Total		13	7	20
**(C) Omicron Variant/PBS Matrix**				**RT-LAMP**		
		**Target** **Concentration/** **Reaction**	**Average Ct**	**Positive**	**Negative**	**Total**
rRT-PCR	Positive	250	33.6	6	0	6
		500	32.9	2	0	2
		1000	32.0	6	0	6
		2500	30.6	2	0	2
	Negative	0	0.0	0	4	4
		Total		16	4	20
(D) Omicron Variant/Fecal Matrix *				**RT-LAMP**		
		**Target** **Concentration/** **Reaction**	**Average Ct**	**Positive**	**Negative**	**Total**
rRT-PCR	Positive	250	34.8	6	0	6
		500	34.0	2	0	2
		1000	33.5	6	0	6
		2500	31.4	2	0	2
	Negative	0	0.0	0	4	4
		Total		16	4	20

* Fecal suspension solutions contained approximately 13.8% semi-solid feces (organic matter by weight) in PBS.

## Data Availability

The raw data supporting the conclusions of this article will be made available by the authors upon request.

## Abbreviations

The following abbreviations are used in this manuscript:

SARS-CoV-2	Severe acute respiratory syndrome coronavirus 2
COVID-19	Coronavirus disease 19
LAMP	Loop-mediated isothermal amplification
RT-LAMP	Reverse transcription loop-mediated isothermal amplification
rRT-PCR	Real-time reverse-transcription polymerase chain reaction
dNTP	Deoxynucleoside triphosphate
PBS	Phosphate-buffered saline
N	Nucleocapsid
E	Envelope
NTC	No-template control
Ct	Cycle threshold
LoD	Limit of detection
HCoV-NL63	Human coronavirus NL63
PHEV	Porcine hemagglutinating encephalomyelitis virus
PEDV	Porcine epidermic diarrhea virus
PDCoV	Porcine deltacoronavirus
TGEV	Transmissible gastroenteritis virus
BdCoV	Bottlenose dolphin coronavirus
FCoV	Feline coronavirus
BCoV	Bovine coronavirus
BRSV	Bovine respiratory syncytial virus
BMT	Blinded method testing
Vet-LIRN	FDA’s Veterinary Laboratory Investigation and Response Network
CI	Confidence interval
DSe	Diagnostic sensitivity
DSp	Diagnostic specificity
dUTP	Deoxyuridine triphosphate
UDG	Uracil DNA glycosylase

## Appendix A

**Table A1:** Summary of the primer sequences utilized in the New England Biolabs SARS-CoV-2 Rapid Colorimetric LAMP Assay Kit. Two different primer sets targeting the E1 region within the envelope gene and the N2 region within the nucleocapsid gene were utilized. For each primer set, a forward outer primer (F3), backward outer primer (B3), forward inner primer (FIP), backward inner primer (BIP), forward loop primer (LF), and backward loop primer (LB) were utilized.

Primer	Sequence
E1-F3	TGAGTACGAACTTATGTACTCAT
E1-B3	TTCAGATTTTTAACACGAGAGT
E1-FIP	ACCACGAAAGCAAGAAAAAGAAGTTCGTTTCGGAAGAGACAG
E1-BIP	TTGCTAGTTACACTAGCCATCCTTAGGTTTTACAAGACTCACGT
E1-LF	CGCTATTAACTATTAACG
E1-LB	GCGCTTCGATTGTGTGCGT
N2-F3	ACCAGGAACTAATCAGACAAG
N2-B3	GACTTGATCTTTGAAATTTGGATCT
N2-FIP	TTCCGAAGAACGCTGAAGCGGAACTGATTACAAACATTGGCC
N2-BIP	CGCATTGGCATGGAAGTCACAATTTGATGGCACCTGTGTA
N2-LF	GGGGGCAAATTGTGCAATTTG
N2-LB	CTTCGGGAACGTGGTTGACC
